# Hyaluronic Acid-Conjugated PLGA Nanoparticles Alleviate Ulcerative Colitis via CD44-Mediated Dual Targeting to Inflamed Colitis Tissue and Macrophages

**DOI:** 10.3390/pharmaceutics14102118

**Published:** 2022-10-05

**Authors:** Shwe Phyu Hlaing, Jiafu Cao, Juho Lee, Jihyun Kim, Aruzhan Saparbayeva, Dongmin Kwak, Hyunwoo Kim, Seonghwan Hwang, Hwayoung Yun, Hyung Ryong Moon, Yunjin Jung, Jin-Wook Yoo

**Affiliations:** 1 College of Pharmacy, Pusan National University, Busan 46241, Korea; 2 State Key Laboratory for Functions and Applications of Medicinal Plants, Guizhou Medical University, Guiyang 550014, China; 3 The Key Laboratory of Chemistry for Natural Products of Guizhou Province and Chinese Academy of Sciences, Guiyang 550014, China

**Keywords:** ulcerative colitis therapy, inflamed tissue targeting, macrophage targeting, nanoparticle, hyaluronic acid

## Abstract

Although various local anti-inflammatory therapies for ulcerative colitis have been developed, rapid drug elimination from inflamed colitis tissue and off-target side effects reduce their therapeutic efficacy. In this study, we synthesized curcumin (Cur)-loaded hyaluronic acid (HA)-conjugated nanoparticles (Cur-HA-PLGA-NPs) that target inflamed colitis tissue via HA-CD44 interaction with resident colonic epithelial cells and subsequently target activated macrophages for ulcerative colitis therapy. The synthesized spherical Cur-HA-PLGA-NPs showed physicochemical properties similar to those of non-HA-conjugated Cur-PLGA-NPs. HA-PLGA-NPs exhibited selective accumulation in inflamed colitis tissue with minimal accumulation in healthy colon tissue. HA functionalization enhanced targeted drug delivery to intestinal macrophages, significantly increasing HA-PLGA-NP cellular uptake. Importantly, the rectal administration of Cur-HA-PLGA-NPs exhibited better therapeutic efficacy than Cur-PLGA-NPs in animal studies. Histological examination revealed that Cur-HA-PLGA-NPs reduced inflammation with less inflammatory cell infiltration and accelerated recovery with re-epithelialization signs. Our results suggest that Cur-HA-PLGA-NPs are a promising delivery platform for treating ulcerative colitis.

## 1. Introduction

Ulcerative colitis (UC) is a chronic disorder characterized by inflammatory gastrointestinal tract (GIT) relapse, especially in the rectum and colon [1]. The annual UC incidence has increased over the past few decades, as UC is clinically challenging to cure and is characterized by high morbidity [2]. Although the etiology of UC remains unclear, it is closely related to immune dysfunction, including inappropriate intestinal inflammatory activation of cells such as macrophages, T cells, and dendritic cells [3]. One of the established UC treatment strategies for controlling abnormal inflammatory cell activation involves the administration of anti-inflammatory and immunosuppressive drugs in oral and rectal dosage forms [4]. However, long-term administration with frequent dosing is required to achieve therapeutic efficacy and severe drug-related side effects can occur [5,6,7]. 

Targeted drug delivery systems to inflamed colitis tissues have been developed for UC therapy to improve drug absorption at inflammation sites, obtain optimum treatment efficacy with lower dosage, and avoid undesired adverse effects. Among them, nanoparticle (NP)-based drug delivery systems have received extensive attention in UC therapy because of their potential to enhance drug accumulation in inflamed colitis tissues. Most studies have been conducted by modifying the physicochemical properties of NPs, including their size and charge, to enhance passive accumulation in the inflamed area [8,9,10]. However, due to the pathophysiological changes in UC, passively accumulated drugs can be easily eliminated from the GIT through diarrhea and mucus clearance [11,12] before reaching the immune cells that reside abundantly in the inflamed colon lamina propria. Therefore, passive accumulation, which can only increase the local drug concentration for a short period in inflamed colitis tissues, is insufficient for effective UC treatment. 

Active targeting enables the direct delivery of therapeutic drugs to colonic epithelial and immune cells, playing critical roles in UC. Due to increased cellular uptake efficiency and sufficient intracellular drug internalization [13], active-targeting drug delivery systems can overcome rapid elimination from inflamed colitis tissues and off-target effects. Macrophages are essential components of the innate immune system in the gut that maintain tissue homeostasis through immunomodulatory effects on inflammatory responses. Activated macrophages critically contribute to UC development by secreting large amounts of pro-inflammatory cytokines, including interleukin-6 (IL-6), IL-12, and tumor necrosis factor-alpha (TNF-α) [14]. Furthermore, various transmembrane receptors such as CD44 [15,16,17,18,19,20], folate (FA) [21,22], mannose receptors [23,24,25,26] and CD98 [27,28], are overexpressed not only on the inflamed colitis tissue but also on activated macrophage surfaces, providing potential binding sites for active-targeting systems. Therefore, macrophages have been recognized as an essential target in UC treatment. Many approaches have been used to actively target activated macrophages via overexpressed receptors using ligands such as folate, mannose, or chondroitin sulfate, showing significant efficacy via receptor-mediated endocytosis [29,30,31]. 

However, due to the presence of these receptors on normal cells throughout the GIT, the administration route of the liganded active-targeting delivery system remains a notable issue. Although patients prefer the oral route of administration because of its convenience and improvement in patient compliance, the risk of non-specific binding of liganded carriers throughout the GIT hinders its use for the treatment of UC that is commonly occurs in the rectum and lower parts of the distal colon. In contrast, the rectal route can skip passage through the upper GI tract and small intestine, providing a high concentration in the colon. Rectal dosage in the form of enema and suppositories is the first line of treatment for mild to moderate left-sided colitis before being combined with oral therapy for extensive colitis [32]. However, the large volume and frequent dosing via the rectal route can lead to patient discomfort and urgency. To meet the challenges associated with non-specific binding and to reduce the dose in terms of volume, rectal administration appears to be a reasonable alternative for a liganded active-delivery system that delivers drugs directly to the target cells, producing therapeutic efficacy at low and less frequent doses.

Therefore, a drug delivery system that can selectively target inflamed colitis tissue and subsequently target intestinal activated macrophages with a targeting ligand, thereby increasing intracellular drug concentration via receptor-mediated endocytosis using the rectal route, will be a novel approach for UC therapy. To address these properties, hyaluronic acid (HA) has emerged as a potent material over other natural polysaccharides that have been utilized for active targeting, owing to its excellent biocompatibility and mucoadhesive properties [33]. It has been approved for use in various routes of administration [34]. Additionally, HA is a native ligand for the CD44 receptor that is overexpressed on inflamed colonic epithelial cells and activated macrophages in UC patients. Many biological functions, including cell migration and adhesion, rely on HA–CD44 interaction. Moreover, HA has been used as an active targeting ligand for modifying nanocarriers to enhance cellular uptake of drugs or nanocarriers via CD44-mediated endocytosis. For example, Wang et al. developed amphiphilic micelles using hyaluronic acid-cystine-PLGA (HA-SS-PLGA) copolymer as a drug carrier to actively target overexpressed CD44 receptors of tumor cells for cancer therapy [35]. Hybrid hyaluronic acid/chitosan (HA/CS) nanoparticles were developed by Lu et al. as non-viral gene delivery vectors that are capable of transferring exogenous genes into primary chondrocytes for osteoarthritis treatment [36]. 

Based on these findings, we hypothesize that HA conjugated with poly (lactic-co-glycolic acid) (PLGA) NPs would selectively target inflamed colitis tissue via HA–CD44 interaction with resident colonic epithelial cells and then target activated macrophages to enhance cellular uptake of the encapsulated drug by the target cells. We chose PLGA as the polymer due to its favorable properties such as biodegradability, biocompatibility, and low toxicity [37,38]. In this study, curcumin (Cur), a natural hydrophobic polyphenolic compound that shows potential anti-inflammatory effects by binding to the nuclear receptor peroxisome proliferator-activated receptor gamma (PPAR-γ) [39], was encapsulated within NPs to evaluate the therapeutic efficacy of the delivery system via CD44-mediated endocytosis. First, we fabricated an HA-conjugated PLGA polymer. Then, Cur-loaded NPs were prepared. After confirming successful NP synthesis, physicochemical properties, in vitro release pattern, and in vivo NP accumulation in the inflamed colitis tissues were assessed. Additionally, the activated macrophage-targeting capacity was visualized using confocal laser scanning microscopy (CLSM). Finally, the therapeutic efficacy of Cur-HA-PLGA-NPs was examined using a dextran sulfate sodium (DSS)-induced colitis mouse model in terms of disease activity index (DAI), histopathological analysis (hematoxylin and eosin staining), immunostaining, and comparison of pro-inflammatory cytokine levels with that of control Cur-PLGA-NPs.

## 2. Materials and Methods

### 2.1. Materials

Poly(D,L-lactide-co-glycolide) AP082 (DL:GA 50:50) with a molecular weight (MW) of 25–35 kDa and an acid end cap was purchased from PolySciTech (Akina, Inc., West Lafayette, IN, USA). Cur, polyvinyl alcohol (PVA, MW: 30–70 kDa), 4-dimethyl aminopyridine (DMAP), 4′,6-diamidino-2-phenylindole dihydrochloride (DAPI), and Nile red were purchased from Sigma-Aldrich (St. Louis, MO, USA). HA (oligo-HA, MW: 0.5–10.1 kDa) was purchased from SK Bioland (Cheonan, South Korea). 1-Ethyl-3-(3-[dimethylamino] propyl) carbodiimide hydrochloride (EDC) was purchased from Dojindo Laboratories (Kumamoto, Japan). 11-Chloro-1,1-di-n-propyl-3,3,3′,3′-tetramethyl-10,12-trimethyleneinda-tricarbocyanine iodide (IR-780) and dimethyl sulfoxide (DMSO)-d6 were purchased from Alfa Aesar (Haverhill, MA, USA). N-Hydroxysulfosuccinimide (NHS) and hematoxylin solutions were purchased from Wako Pure Chemical (Osaka, Japan). Eosin Y solution was purchased from Daejung Chemicals & Metals Co. Ltd. (Shiheung, South Korea). Flamma^®^ 648 NHS ester was purchased from BioActs (Incheon, South Korea). All the other materials and solvents used were of the highest analytical grade.

### 2.2. Synthesis of HA-Conjugated PLGA Polymer (HA-PLGA)

PLGA conjugated with a targeting ligand, HA, was synthesized using the carbodiimide-mediated esterification method. The PLGA carboxylic group (–COOH group) was pre-activated using EDC/NHS as described in a previous study [40]. PLGA (1 g), EDC (0.065 g), and NHS (0.04 g) were completely dissolved in 20 mL dichloromethane (DCM). The mixture was then incubated with gentle rotation for 24 h at 25 °C. Since EDC and NHS are soluble in methanol, unreacted EDC and NHS were removed by adding an excessive amount of methanol. Activated PLGA, which is insoluble in methanol, was precipitated, washed three times with methanol, collected, vacuum-dried, and stored at −20 °C for future uses. To conjugate the HA hydroxyl (–OH) group with PLGA-NHS, 1 g of PLGA-NHS and 0.3 g of HA were dissolved in 20 mL of DMSO containing 0.033 g of DMAP. The conjugation step was carried out by stirring the mixture at 25 °C for 24 h. Finally, HA-conjugated PLGA polymer (HA-PLGA) was precipitated, and unconjugated HA was washed with double-distilled water and collected via sedimentation at 20,000× *g* for 30 min. The pelleted HA-PLGA was washed three times with double-distilled water, freeze-dried, and stored at −20 °C. 

### 2.3. Preparation of NPs

Cur-loaded NPs were fabricated with biodegradable PLGA or HA-PLGA polymers using a simple oil-in-water (o/w) emulsification solvent evaporation method. PLGA or HA-PLGA (100 mg) was dissolved completely in 2 mL dichloromethane with Cur (10 mg) at a drug to polymer ratio of 1:10. This solution was emulsified in 20 mL of ice-cold 1% *w*/*v* polyvinyl alcohol solution using a probe sonicator (KFS-300N ultrasonic, Korea Process Technology, Seoul, South Korea) with an amplitude of 50% for 3 min in an ice bath. The sonicated emulsion was then diluted with 15 mL of distilled water, and the organic solvent was allowed to evaporate via magnetic stirring at 550 rpm under a fume hood. The NPs were collected by centrifugation at 20,000× *g* for 30 min at 4 °C and washed three times with double-distilled water, freeze-dried, and stored at −20 °C for subsequent experiments. IR-780 (1% *w*/*w*) and Nile Red (0.5% *w*/*w*) were used for in vivo imaging system (IVIS) and CLSM studies, respectively, instead of Cur.

### 2.4. Characterization of HA-conjugated PLGA Polymer (HA-PLGA)

The synthesized HA-PLGA polymer was characterized using proton nuclear magnetic resonance spectroscopy (^1^H-NMR) in DMSO-d6 at 500 MHz using a Varian Unity Inova 500 spectrometer (TOSO, Tokyo, Japan). 

### 2.5. Morphology of NPs

The surface morphology of Cur-loaded HA-PLGA NPs (Cur-HA-PLGA-NPs) and Cur-loaded PLGA NPs (Cur-PLGA-NPs) was analyzed by field emission scanning electron microscopy (SEM) and transmission electron microscopy (TEM). For the SEM images, NPs were suspended in distilled water, dropped on a carbon tape, and air-dried in a desiccator. The samples were then coated with platinum for 5 min in a vacuum and viewed using SEM (SUPRA 25, Carl Zeiss, Jena, Germany) at an acceleration voltage of 5.00 kV. For TEM images, NPs dispersed in double-distilled water were placed in appropriate amounts on copper grids with films, dried for 15 min, and observed using TEM (H-7600-Hitachi, Tokyo, Japan). 

### 2.6. Size and Size Distribution Analysis of NPs

The hydrodynamic particle size, polydispersity index (PDI), and zeta potential of Cur-HA-PLGA-NPs and Cur-PLGA-NPs were measured via dynamic light scattering (DLS) using a Zetasizer Nano ZS90 (Malvern Instruments, Worcestershire, UK) after they were dispersed in double-distilled water. Measurements were performed in triplicate for each batch, and the mean and standard deviation (SD) were calculated. 

### 2.7. Determination of Loading Capacity (%), Encapsulation Efficiency (%), and In Vitro Release Pattern

Using an established method, the Cur content in each formulation was determined by measuring the amount of encapsulated Cur in the NPs using a high-performance liquid chromatography (HPLC) system [9]. The HPLC system used in this study was an LC-20AT (Shimadzu, Tokyo, Japan) equipped with an autosampler processor, SPD-20A ultraviolet (UV) detector, and Luna C18 column (5 m, 150 mm × 4.6 m, Phenomenex, Torrance, CA, USA). The wavelength was set to 306 nm when operating the UV detector. The mobile phase was a mixture of acetonitrile and 0.1% trifluoroacetic acid (40:60) at a flow rate of 2 mL/min and sample injection volume of 10 μL. In detail, 5 mg of NPs were dissolved in 1 mL acetonitrile, sonicated in a bath and then transferred to an HPLC autosampler processor. A standard solution of Cur in acetonitrile was used to generate the standard calibration curve. The standard curve for Cur in acetonitrile was evaluated, and the areas under the peaks and concentrations were linearly correlated within a concentration range of 0.01–100 μg/mL (R = 1). Each sample was prepared in triplicate, and the loading capacity and encapsulation efficiency were calculated using Equations 1 and 2 [41].

(1) $$\mathrm{Loading}\;\mathrm{capacity}\;\left(\%\right)=\frac{\left(\mathrm{Amount}\;\mathrm{of}\;\mathrm{curcumin}\;\mathrm{in}\;\mathrm{nanoparticles}\right)}{\left(\mathrm{Amount}\;\mathrm{of}\;\mathrm{nanoparticles}\right)}\times 100$$

(2) $$\mathrm{Encapsulation}\;\mathrm{efficiency}\;\left(\%\right)=\frac{\left(\mathrm{Amount}\;\mathrm{of}\;\mathrm{curcumin}\;\mathrm{in}\;\mathrm{nanoparticles}\right)}{\left(\mathrm{Amount}\;\mathrm{of}\;\mathrm{curcumin}\;\mathrm{initially}\;\mathrm{added}\right)}\times 100$$

In vitro Cur-PLGA-NP and Cur-HA-PLGA-NP release patterns were evaluated using the dialysis-bag method. Briefly, 20 mg of NPs was dispersed in 2 mL of phosphate-buffered saline (PBS, pH 7.4) and sealed in a dialysis bag (MW: 30,000 Da). The bag was then suspended as a sink in 100 mL of releasing buffer (PBS) with constant stirring at 100 rpm at 37 °C in a closed bottle. A total of 1% Tween 80 was added to the release buffer to facilitate Cur dissolution. At predetermined time intervals, 200 μL of PBS was withdrawn from the bottle, and the amount of released Cur was determined using HPLC, as described above. The bottle was refilled with the same amount of fresh PBS to maintain the sink condition.

### 2.8. Animal Studies

Animal experiments were performed according to the Pusan National University Institutional Animal Care and Use Committee (PNU-IACUC) regulations on 24 February 2021 (approval number: PNU-2021-2893). Male imprinted control region (ICR) mice (7 weeks old and weighing 30–35 g) were used as animal models. Food and water were provided ad libitum, and the animals were maintained at 25 ± 3 °C in a 12 h light/dark cycle for 1 week before experimentation to facilitate their adaptation. 

#### 2.8.1. Induction of DSS-Induced Colitis in Mice

Except in healthy control mice, colitis was induced in all mice by oral administration of 5% DSS in distilled water ad libitum for 7 days. During the induction period, the daily DAI, consisting of body weight, stool consistency, and rectal bleeding, was assessed [42].

#### 2.8.2. In Vivo Tissue Accumulation of NPs on Healthy and Inflamed Colitis Tissue of Mice 

After colitis induction, the mice were anesthetized with isoflurane. The same amount of 1% IR-780 dye-loaded HA-PLGA-NPs and PLGA-NPs dispersed in distilled water was administered intrarectally. For comparison, healthy mice were also administered IR-780 NPs intrarectally. After 2 h, the mice were euthanized, and colon sections from the cecum to the anus were harvested. The colon sections were observed using an IVIS (FOBI; Neoscience, Suwon, Korea) before rinsing the luminal content with cold PBS. As described before, Nile red-loaded NPs were used for confocal examination in both healthy and colitis mice. The luminal content was rinsed thoroughly three times with a cold PBS solution to avoid detection of NP mucosal adhesion. The whole colon was rolled using the Swiss-rolling technique, and the colon tissue was fixed in 10% formalin solution for 24 h. The fixed colon tissues were then embedded in a cryostat mold and cut into 5 μm-thick sections using a cryotome (Leica CM1860, Leica Microsystems, Wetzlar, Germany). The prepared colon tissue slides were stained with DAPI for 5 min at room temperature. Finally, the NP accumulation in the inflamed colon tissue was observed using a CLSM (ZEISS LSM 800, Carl Zeiss, Oberkochen, Germany) equipped with a 40× objective lens with two channels (Nile red and DAPI) using a detection wavelength of 565–700 nm and 400–600 nm, respectively.

#### 2.8.3. In Vivo NP Uptake by Activated Macrophages of Inflamed Colitis Tissue 

As mentioned in Section 2.8.2, DSS-induced colitis mice were anesthetized with isoflurane, and the same amounts of Nile red-loaded PLGA and HA-PLGA NPs were administered intrarectally. After 2 h of NP administration, the mice were euthanized, and colon sections from the cecum to the anus were harvested. The luminal content was rinsed three times with cold PBS thoroughly to avoid the detection of fluorescence in the fecal content and mucosal NP adhesion. The colon tissues were fixed in 10% formalin solution for 24 h. The fixed colon tissues were then embedded in a cryostat mold and cut into 5 μm-thick colon tissue sections using a cryotome. Fluorescence immunostaining of the sections was performed according to the manufacturer’s protocol to detect macrophages. The sections were first incubated with 5% BSA solution (blocking reagent) for 1 h at room temperature; sections were then incubated with F4/80 monoclonal antibody (BM*) conjugated with Alexa Fluor 488 (1:100 dilution in 5% BSA solution) overnight at 4 °C. The sections were washed three times with PBS, and the cell nuclei were stained with DAPI for 5 min at room temperature. All the steps were performed under dark conditions to prevent the loss of fluorescence. Finally, sections were mounted with mounting reagent, and NP uptake by the macrophages in the colon tissue was observed with a CLSM (ZEISS LSM 800, Carl Zeiss, Oberkochen, Germany) equipped with a 63× objective lens through three channels, namely Nile red, AF488, and DAPI, using detection wavelengths of 565–700, 410–570 nm, and 400–600 nm, respectively.

#### 2.8.4. Evaluation of Colitis Severity via DAI and Survival Rate

The clinical progression of colitis was monitored daily by assessing each mouse’s DAI score [43]. The DAI score combines weight loss (compared to the initial body weight), stool consistency, and rectal bleeding. Each category is scored on a scale of 0–4. For weight loss, 0 points for no weight loss, 1 point for 1–5%, 2 points for 5–10%, 3 points for 10–20%, and 4 points for >20% weight loss. Stool consistency was scored as 0 for normal stool, 2 for loose stool, and 4 for diarrhea; for bleeding, 0 for no blood, 2 for positive findings, and 4 for gross bleeding. Each group’s survival rate (%) was evaluated daily during the treatment period.

#### 2.8.5. Evaluation of Colon Length, Colon Weight/Length Ratio, and Spleen Weight

On the last day of the experiment (day 14), the animals were euthanized, and the entire colon from the cecum to the anus was resected. After measuring the colon length, the colon was opened longitudinally, and the luminal content was rinsed with cold PBS. Excess PBS on the colon was removed by gentle contact with KIMTECH wipers. The wet colon’s weight was measured, and the calculated colon weight/length ratio was determined as one of the indications of colonic inflammation [44].

#### 2.8.6. Therapeutic Efficacy in a DSS-induced Colitis Mice Model

The male ICR mice (7 weeks old) were divided into four groups (healthy control, colitis control, Cur-PLGA-NP-treated, and Cur-HA-PLGA-NP-treated), with eight mice in each group. Colitis was induced in the mice by administering 5% (*w*/*v*) DSS in drinking water. The drug-treated animal groups received an equal intrarectal dose of Cur (15 mg/kg) once every 2 days for 1 week in the form of suspended NPs in distilled water. The healthy control and colitis control groups received distilled water instead of NPs, and the clinical progression of colitis was monitored daily.

#### 2.8.7. Histological Assessment of Colitis

Colitis severity was also evaluated by histological examinations [45]. The collected colon samples were fixed in 10% formalin for 24 h and embedded in paraffin blocks. Then, 5 μm-thick colon tissue sections were cut with a microtome (Shandon, Pittsburgh, PA, USA). A cross-section of the colon was stained with hematoxylin and eosin and observed under a light microscope (Olympus C, Tokyo, Japan) to determine the colon morphology, epithelial injury, and degree of inflammation [46]. 

#### 2.8.8. Infiltration of Macrophages in Colitis Tissue

To determine macrophage infiltration in the colon tissue, colon cross-sections collected on the last day of the experiment were prepared for fluorescence immunostaining. Then, 5 μm-thick colon tissue sections were prepared as described earlier. After deparaffinization and rehydration of the colon tissue sections, fluorescence immunostaining of the sections was performed according to the manufacturer’s protocol described in Section 2.8.2. Finally, the sections were mounted with a mounting reagent and observed with a CLSM (ZEISS LSM 800, Carl Zeiss, Oberkochen, Germany) equipped with a 40× objective lens with two channels, namely AF488 and DAPI, using detection wavelengths of 410–570 nm and 400–600 nm, respectively.

#### 2.8.9. Quantification of Pro-inflammatory Cytokines via ELISA

The concentrations of pro-inflammatory cytokines, including IL-6 and TNF-α, in the resected mouse colon tissues from the experimental groups were evaluated using Mouse IL-6 ELISA MAXTM Deluxe Set and Mouse TNF-α ELISA MAXTM Standard Set (Biolegend, San Diego, CA, USA), respectively. For sample preparation, frozen colon tissue specimens were homogenized in a lysis buffer solution (10 mL of 1 M tris-hydrochloric acid adjusted to pH 8.0) containing a protease inhibitor cocktail. When the colon specimens were homogenized, the solution was centrifuged for 5 min at 3300× *g*. The supernatant and pro-inflammatory cytokine concentrations were then assayed using an ELISA kit, according to the manufacturer’s instructions.

#### 2.8.10. Statistical Analysis

Data were expressed as mean ± SD. Statistical analyses of all in vitro and in vivo data were performed using two-way and one-way ANOVA followed by the Bonferroni test in GraphPad Prism 5.0 (GraphPad Software, Inc., LA Jolla, CA, USA). *p*-values < 0.001 were considered statistically significant.

## 3. Results and Discussion

### 3.1. HA-PLGA Polymer Characterization

**Figure 1:**
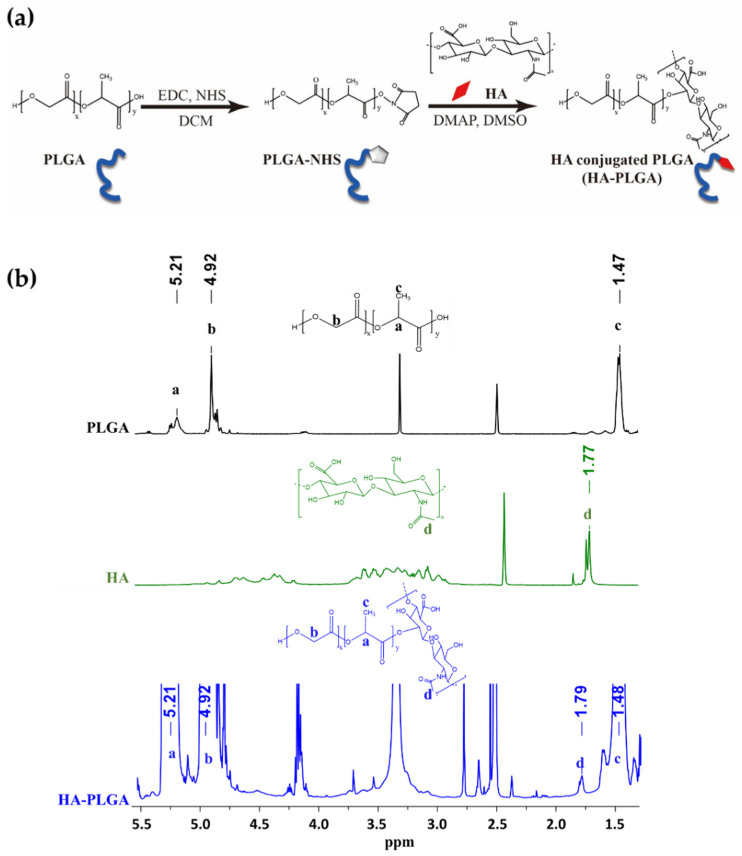
Synthesis and characterization of hyaluronic acid-conjugated PLGA polymer (HA-PLGA). (**a**) HA-PLGA synthesis scheme. (**b**) ^1^H-NMR spectrum of PLGA, HA, and HA-PLGA in DMSO-d6. PLGA: poly(D,L-lactide-co-glycolide); HA: hyaluronic acid; EDC: N-(3-dimethylaminopropyl)-N’-ethyl carbodiimide hydrochloride; NHS: N-hydroxy-succinimide; DCM: dichloromethane; DMAP: 4-dimethylaminopyridine; DMSO: dimethyl sulfoxide.

After HA-PLGA was synthesized using the Steglich esterification method between the PLGA-COOH and the HA-OH groups (Figure 1a), ^1^H-NMR was performed to confirm PLGA and HA conjugation. As shown in Figure 1b, doublets (-CH_3_ group; c) of L-lactic acid at 1.4 ppm, multiplets (-CH group; a) of lactic acid at 5.2 ppm, and multiplets (-CH_2_ group; b) of glycolic acid at 4.9 ppm of PLGA polymer were observed in the ^1^H-NMR spectra of HA-PLGA. In addition, both the HA and HA-PLGA spectra presented a peak at 1.7 ppm which represents the acetamido moiety (-CH_3_ group; d) of N-acetyl-D-glucosamine which indicated the successful conjugation of polymer. One PLGA molecule which possess more than 10 repeating units of lactic and glycolic acids had only one –COOH group to conjugate with one HA molecule. Since HA has a smaller MW than PLGA polymer, the peak that represented HA was much smaller than that of PLGA in the HA-PLGA spectra. Moreover, HA-PLGA has physicochemical properties similar to those of PLGA polymers, such as hydrophobicity.

### 3.2. Fabrication and Characterization of PLGA-HA NPs

**Figure 2:**
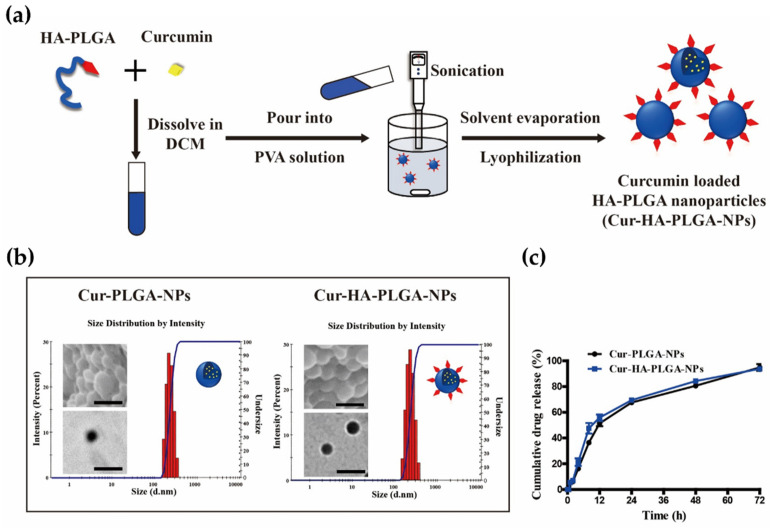
(**a**) Schematic illustration of the preparation of curcumin-loaded HA-conjugated PLGA nanoparticles (Cur-HA-PLGA-NPs). DCM: Dichloromethane; PVA: Polyvinyl alcohol. (**b**) SEM, TEM, and size distribution intensity of Cur-PLGA-NPs and Cur-HA-PLGA-NPs. Scale bar represents 200 nm. (**c**) In vitro release pattern of curcumin from Cur-PLGA-NPs and Cur-HA-PLGA-NPs.

The current study utilized the anti-inflammatory drug Cur, which regulates innate immune cell migration, such as that of macrophages, from the peripheral circulation to inflammatory sites, exerting beneficial effects in experimental colitis [47,48,49]. Since Cur is highly hydrophobic, has low solubility, and rapidly undergoes hydrolytic degradation above neutral pH [50], a large amount and frequent dosing are required to achieve a therapeutic effect against inflammatory diseases. Hydrophobic Cur was encapsulated within NPs using HA-PLGA and PLGA polymers through a simple oil-in-water emulsion solvent evaporation method (Figure 2a). All fabrication steps were performed under dark conditions because Cur is photodegradable in organic solvents [51]. After washing and lyophilization, the morphology of the NPs was observed using SEM and TEM. Both Cur-PLGA-NPs and Cur-HA-PLGA-NPs had a spherical morphology without Cur powder around the NPs (Figure 2b). HA was observed outside the NPs in the TEM image of the light-darker region surrounding the strong contrast core region. The physicochemical properties of Cur-PLGA-NPs and Cur-HA-PLGA-NPs were characterized (Table 1). The average size of Cur-PLGA-NPs and Cur-HA-PLGA-NPs measured by Zetasizer was 225 nm and 234.4 nm, respectively. Both NPs revealed a narrow size distribution using the PDI (0.03 and 0.02 for Cur-PLGA-NPs and Cur-HA-PLGA-NPs, respectively) and particle size distribution histograms of NPs (Figure 2b). Since HA-PLGA polymer has similar physicochemical properties to PLGA, including solubility, the hydrophobic Cur was encapsulated within PLGA and HA-PLGA polymers without significant differences in loading capacity (5.3% and 5.8%, respectively) or encapsulation efficiency (58.3% and 63.4%, respectively). Furthermore, the zeta potential (mV) of both NPs showed a negative charge. Cur-PLGA-NPs and Cur-HA-PLGA-NPs exhibited zeta potentials of −13.6 mV and −14.6 mV, respectively.

According to the in vitro release test, neither NP showed significant differences in their drug release profiles. After 48 h of incubation in the releasing media at pH 7.4, 80.65% and 84.33% Cur were released into the releasing media from Cur-PLGA-NPs and Cur-HA-PLGA-NPs, respectively (Figure 2c).

### 3.3. In Vivo Imaging of Accumulation of NPs on the Healthy and Inflamed Mice Colon 

**Figure 3:**
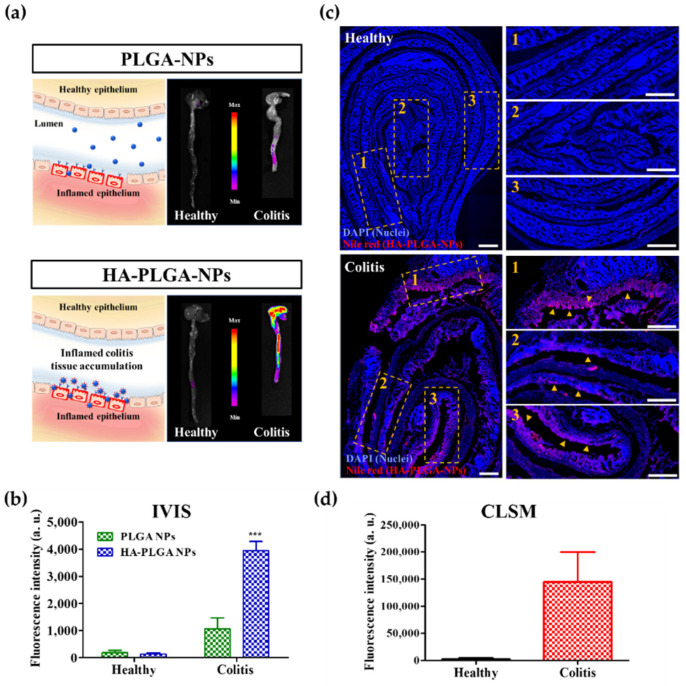
In vivo accumulation of nanoparticles (NPs) in healthy and inflamed colitis tissue of mice after incubation with IR-780-loaded PLGA-NPs and HA-PLGA-NPs for 2 h. (**a**) Schematic illustration with representative in vivo imaging system (IVIS) images indicating NP accumulation in healthy and inflamed colon (colitis). (**b**) Fluorescence intensity observed using IVIS. (*** *p* < 0.001) (**c**) Confocal laser scanning microscope (CLSM) images of Nile red-loaded HA-PLGA-NPs in healthy and inflamed colitis tissue after 2 h of intrarectal administration. The blue color represents DAPI nuclear staining, and the red color represents Nile red-loaded NPs. The orange rectangles (1, 2, and 3) with the orange arrowheads indicate the accumulation of Nile red-loaded HA-PLGA-NPs in the epithelium of the inflamed colon. (**d**) Fluorescence intensity observed using CLSM. “a.u.” indicates fluorescence arbitrary units.

The mucus layer is a protective system for the GIT epithelium [11]. Due to UC pathogenesis, increased mucus secretion and a penetrable inner mucus layer, which allowed the sedimentation of beads to the epithelium, were observed in the colon of patients with active UC [52]. Although the mucus layer preferentially traps foreign materials through hydrogen bonds, hydrophobic forces, or electrostatic interactions, entrapped materials are excreted from the GIT via mucus clearance [53,54]. Additionally, drug accumulation in the inflamed colitis tissue still limits therapeutic efficiency due to the low cellular uptake of the targeted cells and insufficient intracellular drug release. Our study investigated HA-PLGA-NP accumulation in inflamed colitis tissues and compared it to that in healthy colon tissues. IR-780-loaded PLGA-NPs were also used as a control to compare the tissue accumulation properties of NPs via HA functionalization. After rectal administration of IR-780-loaded HA-PLGA-NPs for 2 h, a high intensity of IR-780 was detected in the colon tissues of mice with DSS-induced colitis by IVIS (Figure 3a). Since the complete renewal of the mucus layer occurs within 2 h in mice [55], a low IR-780 intensity in the healthy colon was observed after IR-780 NP administration for 2 h, indicating that most of the HA-PLGA-NPs that accumulated on the mucus layer of the healthy control mice were excreted from the GIT via mucus clearance. In contrast, HA-PLGA-NPs with negative charges penetrated the mucus layer and were actively attracted to the inflamed colon epithelium because of the overexpressed CD44 receptors [56]. To investigate NP accumulation in the inflamed colon epithelium, the colon was cut longitudinally; luminal colon contact was washed three times with cold PBS, and the colon samples were prepared by the Swiss roll technique, fixed, and cut into 5 μm-thick colon sections with a cryotome. The cell nuclei were stained with DAPI and observed under CLSM. Nile red fluorescence was not detected in the healthy colon tissue (Figure 3c). In contrast, robust Nile red fluorescence was detected in the colitis mice. According to the CLSM results, HA-PLGA-NPs were observed deep inside the layers of inflamed colitis tissue, depending on the severity of the inflammation (orange rectangle in Figure 3c). These results suggest that HA functionalization improves the selective accumulation of NPs more significantly in an inflamed colitis tissue than in a healthy colon tissue. 

### 3.4. In Vivo CLSM Imaging of Cellular Uptake of NPs by Inflammatory Macrophages 

**Figure 4:**
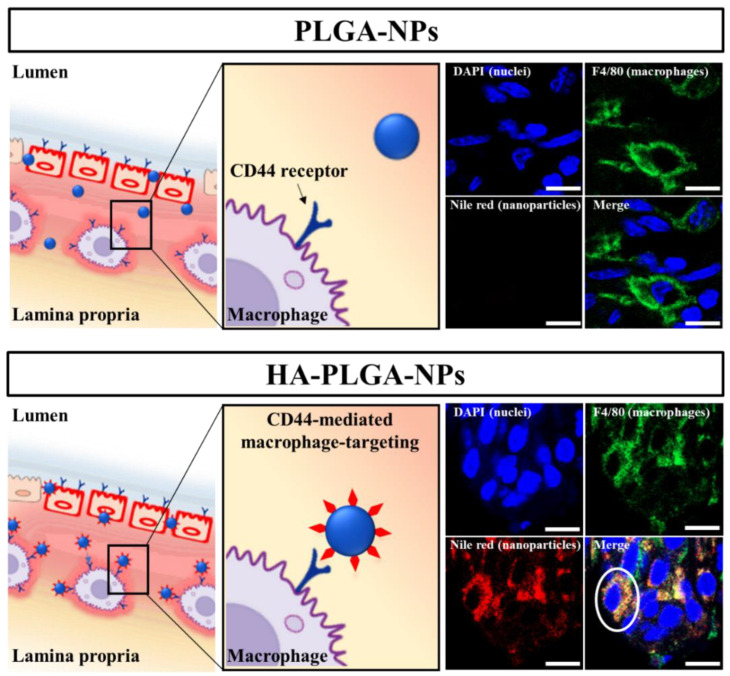
In vivo cellular uptake of NPs by macrophages in a DSS-induced colitis mouse model after 2 h of incubation with Nile red-loaded PLGA and HA-PLGA NPs shown through representative CLSM images from Nile red-loaded PLGA-NPs and HA-PLGA-NP-treated colitis tissues with schematic illustrations. The blue, green, and red colors represent nuclear staining (DAPI), macrophages (F4/80), and Nile red-loaded NPs, respectively. The white circle indicates the accumulation of Nile red-loaded HA-PLGA-NPs in the macrophage of the inflamed colon. The scale bar represents 20 µm.

As intestinal macrophages are essential cells of the innate immune system, they play a vital role in UC severity. The intestinal lamina propria is the most prominent location where macrophages are abundant. The immune system controls the balance between pro-inflammatory and anti-inflammatory factors to maintain tissue homeostasis. When UC occurs, the inflamed epithelial cells stimulate the immune cells to secrete several pro-inflammatory factors, leading to an immune system imbalance, thereby aggravating UC severity. Consequently, the regulation of intestinal macrophages has attracted attention as an essential strategy for treating UC. HA has been understood to bind specifically to CD44 receptors, which are overexpressed on the surfaces of the activated macrophages that infiltrate the inflamed colon [57]. In this in vivo study, we added Nile red-loaded PLGA-NPs as a control to determine how HA functionalization affects the NP internalization by inflammatory cells (macrophages). We rinsed the collected colon tissue samples thoroughly with PBS three times before preparing them to prevent the detection of signals from NPs adhering to the inflamed colon mucus layer. No Nile red signal was detected, indicating that PLGA-NPs were not taken up by F4/80-labeled inflamed colon macrophages (Figure 4). Particles smaller than 10 μm showed higher adherence to inflamed tissues, with thicker mucus layers and ulcerated areas [8]. According to the results, although particles adhered to inflamed tissues, they could not actively target specific cells without a targeting moiety. Conversely, the vigorous intensity of the Nile red fluorescence signal, representing HA-PLGA-NPs, was taken up by macrophages in the inflamed colon (white circle in Figure 4). These results indicate that HA functionalization extensively affects the active targeting efficacy of HA-PLGA-NPs toward specific inflammatory cells (macrophages), enhancing cellular uptake by target cells and intracellular encapsulated drug release, which in turn improve therapeutic efficacy. 

### 3.5. Macroscopic Analysis of Colitis after Treatment with NPs 

**Figure 5:**
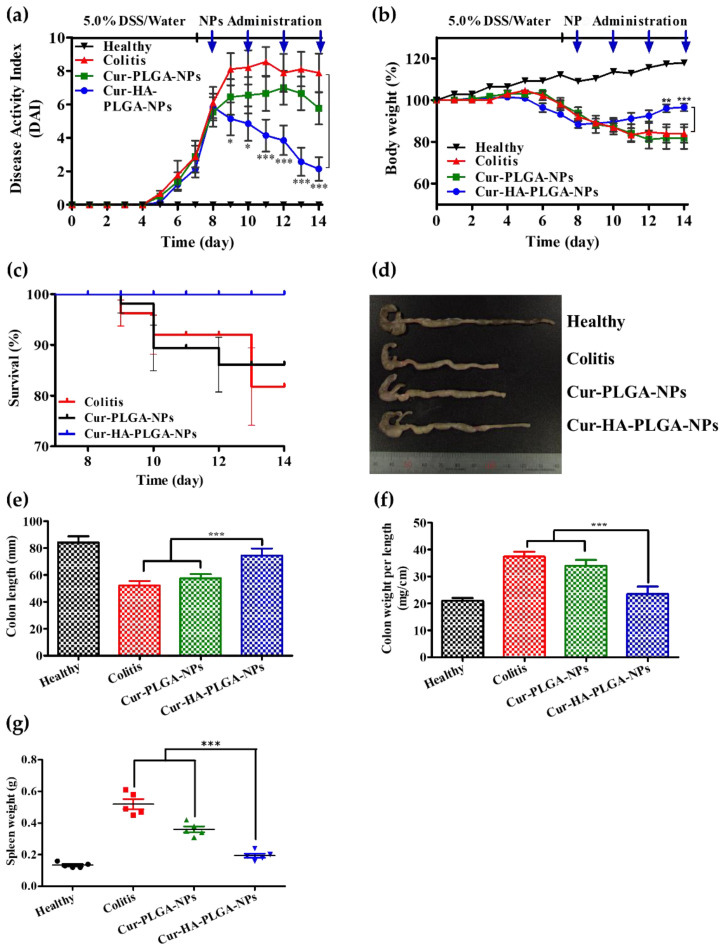
Therapeutic efficacy of Cur-HA-PLGA-NPs and Cur-PLGA-NPs against DSS-induced colitis mice model. (**a**) Disease activity index. (**b**) Body weight changes (%). The blue-headed arrow represents the day of NP administration. (**c**) Survival rate of colitis mice after NP treatment. (**d**) Colon photograph from each experimental mouse group. (**e**) Colon length measurement. (**f**) Measurement of colon weight per length. (**g**) Spleen weight. Statistical comparisons were evaluated between the Cur-HA-PLGA-NP-treated groups with the colitis control group and the Cur-PLGA-NP-treated group (* *p* < 0.05, ** *p* < 0.01, *** *p* < 0.001). *n* = 5.

To study the in vivo Cur-HA-PLGA-NP therapeutic efficacy, the same amount of NPs (Cur 15 mg/kg) was rectally administered to a DSS-induced colitis mouse model. Based on the delayed drug release pattern and in vivo cellular uptake results, the treatment was administered once every 2 days for 1 week. Furthermore, because DSS-induced colitis histopathology in mice resembles that in humans, the DSS model was chosen. DAI values are widely used to indicate colitis severity [58]. According to the DAI results of all studied mouse groups, the healthy control group showed constant DAI values, representing no colitis throughout the study period. In the DSS-induced colitis mouse model, the colitis control group exhibited severe diarrhea and bleeding, indicated by the high DAI values (Figure 5a). In contrast, colitis mice treated with Cur-loaded NPs showed lower DAI values than those in the colitis control group. The Cur-HA-PLGA-NP-treated group had significantly lower DAI values than the Cur-PLGA-NP-treated group. Additionally, differences in the body weights of the mice were evaluated. During the incubation period (1 week), there was no difference in the body weight of the mice among the groups. After colitis induction was initiated via DSS administration, there was a significant decrease in the body weight of the mice in the colitis control, Cur-PLGA-NP-, and Cur-HA-PLGA-NP-treated groups. A faster body weight recovery was observed in the Cur-HA-PLGA-NP-treated group (Figure 5b). Furthermore, the Cur-HA-PLGA-NP-treated group showed a better survival rate than the colitis control and Cur-PLGA-NP-treated groups (Figure 5c). 

DSS-induced colitis with severe diarrhea and bleeding is accompanied by colon length shortening [10]. As an index of colitis, the colon was significantly shortened in the DSS-induced colitis group than in the healthy control. The healthy mice had an average colon length of ~85 mm (Figure 5d,e). In contrast, the colitis control group had a remarkably shortened colon length (~52 mm). Compared with the colitis group, the Cur-PLGA-NP-treated group exhibited no significant difference in colon length (~57 mm), indicating high colitis severity. However, the Cur-HA-PLGA-NP-treated group showed a remarkable improvement in colon length (~74 mm). Additionally, another colitis clinical activity index, the colon weight/length ratio, was evaluated in comparison with each group. The Cur-HA-PLGA-NP-treated group exhibited lower colon weight/length ratio values than the colitis group, indicating colitis recovery (Figure 5f). Although both Cur-loaded NPs showed therapeutic effects in colitis, the same amount of Cur-HA-PLGA-NPs exhibited significantly enhanced therapeutic efficacy compared to that of Cur-PLGA-NPs. The colitis grade and recovery evaluated by DAI, body weight, colon length, and the colon weight/length ratio showed that Cur-HA-PLGA-NPs exhibited significantly better therapeutic effects than Cur-PLGA-NPs. As the spleen is an essential organ of the immune system, spleen weight is used as an inflammation severity marker [59]. The spleen weight results are shown in Figure 5g. The spleen weight increased with increased inflammation. Compared to the healthy group (0.13 g), the colitis control group showed a remarkably increased spleen weight (0.52 g). Decreased spleen weight was observed in both NP groups after alleviating inflammation. However, the Cur-HA-PLGA-NP-treated group exhibited a lower spleen weight (0.19 g) than the Cur-PLGA-NP-treated group (0.36 g). The results demonstrated that Cur-HA-PLGA-NPs ameliorated colitis better than Cur-PLGA-NPs.

### 3.6. Histological Analysis and Immunofluorescence of Colitis after Treatment with NPs 

**Figure 6:**
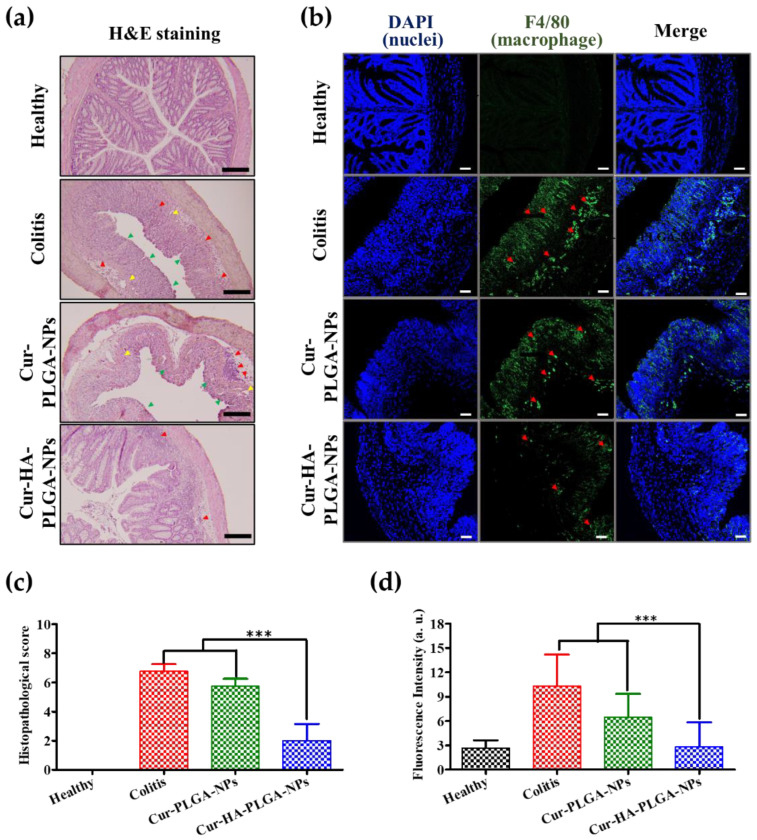
Microscopic assessment of colitis after treatment with Cur-PLGA-NPs and Cur-HA-PLGA-NPs. (**a**) Hematoxylin and eosin staining for microscopic evaluation of the colon sections isolated from healthy control, colitis control, Cur-PLGA-NP-treated, and Cur-HA-PLGA-NP-treated mouse groups. The black scale bar represents 200 µm. The yellow, red, and green arrowheads indicate submucosal edema, infiltration of inflammatory cells, and damaged epithelium, respectively. (**b**) Immunohistochemical assessment of colitis (macrophage infiltration). F4/80 immunostaining images of colon tissue from healthy control, colitis control, Cur-PLGA-NP-treated, and Cur-HA-PLGA-NP-treated mouse groups. The blue, and green colors represent nuclear staining (DAPI), and macrophages (F4/80), respectively. The white scale bar represents 50 µm. The red arrowhead indicates infiltrated macrophages in colon tissue. (**c**) Histopathological score. (**d**) Fluorescence intensity. “a.u.” represents fluorescence arbitrary units. (*** *p* < 0.001). The results are presented as the mean ± S.D.

Histological examination of the colon tissue sections removed from the experimental mice was performed by H&E staining to evaluate the therapeutic potential of Cur-HA-PLGA-NPs for colitis treatment. The colon sections from the healthy control group exhibited no signs of disrupted epithelium, absence of mucosal edema, or inflammatory cell infiltration (Figure 6a). Hence, the colon sections from DSS-induced colitis mice showed marked signs of severe colitis, including epithelium damage, goblet cell depletion, submucosal edema, and inflammatory cell infiltration (neutrophils and macrophages). The Cur-PLGA-NP-treated groups showed no remarkable improvement in colitis compared to the colitis group. Interestingly, the Cur-HA-PLGA-NP-treated group colon sections exhibited pronounced improvements in histological colitis appearance, including signs of re-epithelization and histological features resembling those of healthy colon sections. 

Inflammatory cell infiltration (macrophages and neutrophils) at the site of inflammation that produces pro-inflammatory cytokines, such as IL-6 and TNF-α, is believed to be involved in colitis pathogenesis. An immunofluorescence study of macrophages (F4/80-positive cells) in the colonic mucosa was performed to investigate the relationship between macrophage infiltration and the severity of colitis. The colon tissue sections from healthy mice showed no signs of macrophage infiltration into the mucosa, indicating the absence of colitis (Figure 6b,d). In contrast, increased macrophage infiltration into the epithelium was observed in colon tissue sections from the mice with untreated colitis. Compared with the colitis mice, colon sections from the mice treated with Cur-PLGA-NPs showed reduced macrophage infiltration. Interestingly, the treatment of mice with Cur-HA-PLGA-NPs significantly reduced macrophage infiltration into the colonic mucosa, indicating an improvement in colitis treatment.

### 3.7. Measurement of Pro-inflammatory Cytokine Levels 

**Figure 7:**
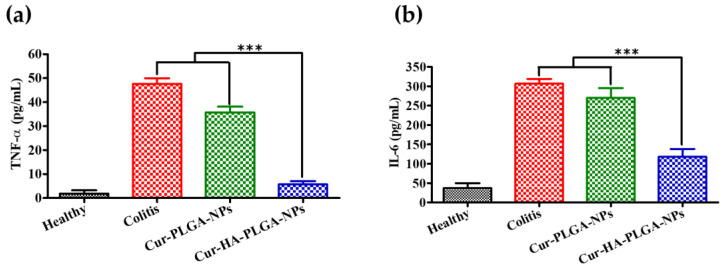
Pro-inflammatory cytokine levels in the healthy control, colitis control, Cur-PLGA-NP-treated, and Cur-HA-PLGA-NP-treated groups. (**a**) TNF-α and (**b**) IL-6 (*** *p* < 0.001). Statistical comparisons were evaluated between the Cur-HA-PLGA-NP-treated and control groups (colitis control and Cur-PLGA-NP-treated groups). Results are expressed as the mean ± SD.

All mice were sacrificed on the last day of the experiment (day 14), and their colon sections were resected for cytokine analysis. IL-6 and TNF-α secreted by neutrophils and macrophages are two key pro-inflammatory cytokines associated with colitis pathogenesis [60]; therefore, blockage of their production is an essential approach to treating colitis [61]. Thus, we confirmed pro-inflammatory cytokine (IL-6 and TNF-α) expression profiles in all experimental groups. The IL-6 and TNF-α levels detected in colon samples were increased in response to colitis induction in mice. However, cytokine levels were significantly lower in the mice colon samples treated with Cur-HA-PLGA-NPs than those treated with Cur-PLGA-NPs and colon samples from untreated colitis mice (Figure 7a,b).

## 4. Conclusions

This study successfully developed Cur-HA-PLGA-NPs for UC therapy in two steps: polymer conjugation and NP preparation. We confirmed that Cur-HA-PLGA-NPs could target inflamed colitis tissue via HA-CD44 interaction with resident colonic epithelial cells and subsequently target activated macrophages using in vivo tissue accumulation and cellular uptake studies. The rectal administration of Cur-HA-PLGA-NPs to a DSS-induced UC mouse model showed enhanced therapeutic efficacy compared to the Cur-PLGA-NP-treated and colitis groups. This result was further supported by the histological appearance revealing that the Cur-HA-PLGA-NP-treated group exhibited rapid recovery from colitis, including signs of re-epithelization and less inflammatory cell infiltration similar to that of the healthy colon. Furthermore, significantly low pro-inflammatory cytokine levels (TNF-α and IL-6) in the Cur-HA-PLGA-NP-treated group indicated colitis alleviation, which is generally high in chronic inflammation. Based on these results, we concluded that Cur-HA-PLGA-NPs prepared in this study may be a promising drug delivery system for treating UC.

## Figures and Tables

**Table 1:** Physicochemical properties of curcumin-loaded nanoparticles. Results are expressed as the mean ± standard deviation (SD) (n = 5).

Formulation	Size (nm)	Polydispersity Index	Loading Capacity (%)	Encapsulation Efficiency (%)	Zeta Potential (mV)
Cur-PLGA-NPs	225.0 ± 3.55	0.03 ± 0.02	5.3 ± 0.3	58.3 ± 3.7	−13.6 ± 0.3
Cur-HA-PLGA-NPs	234.4 ± 1.27	0.02 ± 0.01	5.8 ± 0.3	63.4 ± 3.6	−14.6 ± 0.3

## Data Availability

Not applicable.
